# Skin asepsis protocols as a preventive measure of surgical site infections in dogs: chlorhexidine–alcohol versus povidone–iodine

**DOI:** 10.1186/s12917-018-1368-5

**Published:** 2018-03-14

**Authors:** Luís Belo, Isa Serrano, Eva Cunha, Carla Carneiro, Luis Tavares, L. Miguel Carreira, Manuela Oliveira

**Affiliations:** Centre for Interdisciplinary Research in Animal Health (CIISA) / Faculty of Veterinary Medicine, University of Lisbon, Avenida da Universidade Técnica, 1300-477 Lisbon, Portugal

**Keywords:** Asepsis, Antimicrobial resistance, Chlorhexidine, Dogs, Povidone-iodine, Skin infection, *Staphylococci*, MRSA, MRSP, Pre-surgery

## Abstract

**Background:**

Most of surgical site infections (SSI) are caused by commensal and pathogenic agents from the patient’s microbiota, which may include antibiotic resistant strains. Pre-surgical asepsis of the skin is one of the preventive measures performed to reduce SSI incidence and also antibiotic resistance dissemination. However, in veterinary medicine there is no agreement on which biocide is the most effective.

The aim of this study was to evaluate the effectiveness of two pre-surgical skin asepsis protocols in dogs. A total of 46 animals were randomly assigned for an asepsis protocol with an aqueous solution of 7.5% povidone-iodine or with an alcoholic solution of 2% chlorhexidine. For each dog, two skin swab samples were collected at pre-asepsis and post-asepsis, for bacterial quantification by conventional techniques and isolation of methicillin-resistant species.

**Results:**

Most samples collected at the post-asepsis did not present bacterial growth, both for the animals subjected to the povidone-iodine (74%) or to the chlorhexidine (70%) protocols. In only 9% of the cases a significant bacterial logarithmic reduction was not observed, indicating possible resistance to these agents. Also, the logarithmic reduction of the bacterial quantification from pre- and post-asepsis time, was not statistically different for povidone-iodine (6.51 ± 1.94 log10) and chlorhexidine (6.46 ± 2.62 log10) protocol.

From the 39% pre-asepsis swabs which showed bacterial growth in MRSA modified chromogenic agar medium, only one isolate was identified as *Staphylococcus aureus* and one as *S. epidermidis*. False positives were mainly other staphylococci species, as well as Enterobacteriaceae.

**Conclusions:**

Pre-surgical skin asepsis protocols with povidone-iodine or chlorhexidine showed similar efficacy in the elimination of methicillin resistant bacteria and preventing surgical site infections in dogs undergoing surgery.

## Background

Surgical site infections (SSI) are the most common type of medical-related infection in developing countries, and the second most frequent type in Europe and the United States [1]. These infections remain a significant cause of morbidity and mortality worldwide, being responsible for significant healthcare costs [1, 2]. Since most SSI are caused by microorganisms from the commensal microbiota of the patient, it is important to perform and ensure efficient pre-surgical skin asepsis [3]. With the aim of standardize pre-, intra- and post- surgical procedures to prevent surgical site infections in human medicine, some guidelines were recently developed [1, 2]. The World Health Organization (WHO) as well The Center for Disease Control and Prevention (CDC) recommended the use of alcohol-based agents for the skin asepsis of patients undergoing surgery [1, 2].

In veterinary medicine there is no agreement on which biocide has the greatest efficacy in reducing the skin microbiota, aiming at preventing SSI development [4–6]. In addition, different skin asepsis protocols may be adopted according to the location of surgery in dogs [7–9]. For instance, in bitches undergoing ovariohysterectomy, 0.3% stabilized glutaraldehyde and alcohol (SG + A), 0.3% SG and water, and 4% chlorhexidine gluconate tincture revealed to be equally effective [8].

Due to their commensal and opportunistic nature, staphylococci are the main bacterial agents isolated from surgical site infections in dogs [10], being *Staphylococcus pseudintermedius* the most frequent [11]. *S. pseudintermedius* is commensal in dogs, whereas *S. aureus* is commensal in humans, and the presence of *S. aureus* on dogs have a human origin. It is also important to refer that in the last decade there has been a rapid emergence of infections by methicillin-resistant *S. pseudintermedius* (MRSP) in dogs and cats [3]. There is a great concern about MRSP, which is the main cause of surgical site infections in some regions and its treatment is greatly hampered by the high levels of antimicrobial resistance [12]. Due to its high resistance levels, the incidence of MRSP is an even more relevant problem than methicillin-resistant *S. aureus* (MRSA) [10]. Also, although *S. aureus* is also isolated from SSI, its occurrence is less frequent [10].

To date, studies comparing different pre-surgical skin asepsis protocols with application in veterinary medicine are scarce. Therefore, it is vital to clarify which is the most efficient protocol for preventing bacterial growth and the dissemination of multiresistant pathogenic bacteria, such as MRSP and MRSA.

The aim of this study was to evaluate two pre-surgical skin asepsis protocols as a preventive measure of SSI in dogs, namely an aqueous solution of 7.5% povidone-iodine or an alcoholic solution of 2% chlorhexidine. Povidone-iodine has an excellent immediate antimicrobial effect [13], whereas chlorhexidine is the most used biocide in antiseptic products, being more effective against gram-positive bacteria than gram-negative bacteria, fungi and viruses [14].

## Methods

### Samples

A total of 46 dogs presented for orthopedic or soft tissues surgery in several anatomical regions were included in this study. The group comprised 17 males and 29 females, aged between 7 months and 16.3 years (average ($\overline{X}$) ± standard deviation (δ) = 6.6 ± 4.3 years), and weights ranging between 1.8 kg and 38.2 kg (median ± interquartile range = 15.6 ± 13.0). All dogs included in the study performed pre-surgical exams, and those which laboratory standard tests results deviated from the reference values were automatically excluded. Of the 46 canids, 23 were selected randomly and blindly for the evaluation of the 7.5% povidone-iodine asepsis protocol, and the remaining 23 for the evaluation of the 2% chlorhexidine. From each canid, one skin swab sample was collected before asepsis (t0), and the second post-asepsis (t1).

Post-surgical evaluation of all dogs was performed at 24 h and at day 30 (no implants applied) by the veterinary surgeon, to access the presence of SSI signs.

### Pre-surgical skin asepsis protocols

With the patient already anesthetized with Propofol in lipid emulsion at 10 mg/mL (1–4 mg / kg i.v.), an area of ​​10 × 10 cm was delimited for sampling using a sterile dressing, after which the hair removal and removal of dirt from the surgical site were performed. The first sample (t0) was collected, performing a skin smear of ​​the previously delimited area, with the aid of a swab (swabs with Amies transport medium) (VWR, Alfragide, Portugal). Subsequently, the biocide selected for the group to which the animal was allocated was applied over the area, using sterile 10 × 10 cm compresses.

A group of 23 animals was submitted to the asepsis protocol with an aqueous solution of 7.5% povidone-iodine (Braunol®) (B. Braun Medical, Lda, Queluz de Baixo, Portugal), followed by the application of Braunol® spray, and the other 23 animals were submitted to an asepsis protocol with an alcohol solution of 2% chlorhexidine gluconate (Desinclor® 2%) (Laboratorios Vaza, Madrid, Spain), followed by the application of 70% isopropyl alcohol spray. One minute after application of the biocide, a second smear of the delimited area (t1) was performed.

All swabs collected at the pre-asepsis were placed in test tubes with 1 mL of sterile saline, vortexed, and diluted (10 to 10^− 5^). From each dilution, 100 μL were collected and plated on Brain Heart Infusion agar (VWR, Alfragide, Portugal), a nonspecific enrichment culture medium, and incubated at 37 °C for 48 h. From the initial suspension, 100 μL were also plated on MRSA agar (CONDA laboratories - pronadisa, Madrid, Spain), a selective and differential chromogenic agar medium supplemented with cefoxitin, for the isolation of *S. aureus* and *S. epidermidis* after a 24–48 h incubation at 35 ± 2 °C.

Skin swabs collected at post-asepsis were also placed in test tubes with 1 mL of sterile saline, vortexed, and plated on BHI and MRSA agar.

After incubation, bacterial quantification was performed by determining the colony forming units in BHI agar at 24 h and 48 h, and also at 72 h in in the MRSA medium.

Colonies grown in MRSA agar were isolated on Columbia agar medium + 5% sheep blood (Biomérieux, Linda-a-Velha, Portugal) before microscopic observation after Gram staining and identification. Gram-positive cocci were further evaluated by catalase and potassium hydroxide (KOH) tests, and catalase-positive isolates were identified by API® Staph biochemical galleries (Biomérieux, Linda-a-Velha, Portugal). Gram-positive bacilli were evaluated by catalase and KOH tests (VWR, Lisbon, Portugal), and catalase-positive isolates were identified using API® Coryne. Gram-negative bacilli were submitted to the oxidase (VWR, Lisbon, Portugal) and KOH tests, and oxidase-negative isolates were identified through API® 20E galleries. Finally, gram-positive coccobacillus was plated onto Edwards and Slanetz and Bartley agar medium (VWR, Lisbon, Portugal) for presumptive identification of *Enterococcu*s spp.

### Statistical analysis

The analysis of variance was performed using ANOVA with repeated measures. For statistical purposes, microbial quantifications in BHI culture medium were subsequently converted to base 10 logarithms, using the formula log10(CFU/mL + 1), allowing to include in the analysis all null quantifications at the post-asepsis time. Plates where quantification proved to be uncountable were considered as > 10^9^ CFU / mL (> 9 log10).

## Results

Distribution of dogs in the two groups subjected to the different pre-surgical skin asepsis protocols was similar regarding sex, age and weight, type of surgery, and previous exposure to antibiotic therapy and immunosuppressants (Table 1).

**Table 1:** Distribution of the animals included in this study (*n* = 46)

Canids features	A	B
Males (n)	8	9
Females (n)	15	14
Age (average in years)	6.62	6.65
Weight (average in Kg)	16.23	15.75
Orthopedic Surgery	5	5
Abdominal Surgery	15	9
Non-abdominal Surgery	3	9
Prophylactic antibiotic therapy	23	23

Animals distributed according to gender, age and weight average, type of surgery, prophylactic antibiotic therapy, between the two groups A (pre-asepsis protocol with povidone-iodine) and B (pre-asepsis protocol with chlorhexidine)

All surgically created wounds were clean, according to surgical wound classifications identified by CDC [15].

### Bacterial quantification in BHI agar

Only 26% (6/23) of the animals submitted to the asepsis protocol with povidone-iodine, and 30% (7/23) of the ones submitted to the chlorhexidine protocol presented positive bacterial growth after incubation of the skin swab collected at post-asepsis.

Regarding bacterial load reduction between pre- and post-asepsis, only 4.3% (1/23) of the animals subjected to povidone-iodine and 13% (3/23) subjected to chlorhexidine did not present a significant logarithmic reduction in bacterial quantification.

**Fig. 1:**
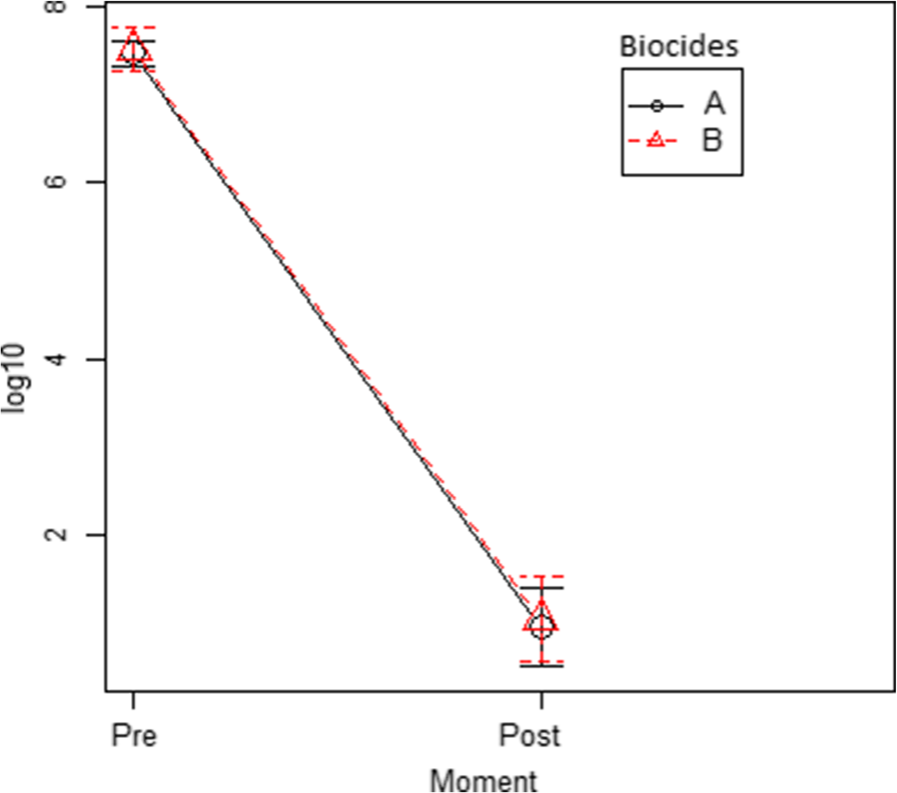
Logarithmic reduction of the bacterial quantification at pre-asepsis and post-asepsis: The log reduction of the bacterial quantification between samples collected at pre-asepsis and post-asepsis using povidone-iodine (A) and chlorhexidine protocols (B)

For the povidone-iodine protocol, the mean bacterial quantification at pre-asepsis was 8.03 × 10^7^ CFU/mL and the log mean was 7.47 log10. The mean bacterial quantification at post-asepsis was 4.35 × 10^7^ CFU/mL and the log mean was 0.95 log10. There was a decrease of 3.68X10^7^ CFU/mL between pre-asepsis and post-asepsis quantifications, and the logarithmic reduction was 6.51 ± 1.94 log10 (mean ± standard deviation) (Fig. 1).

For the chlorhexidine protocol, the mean bacterial quantification at pre-asepsis was 4.0 × 10^8^ CFU/mL, and baseline log mean was 7.51 log10. The mean bacterial quantification at post-asepsis was 4.42 × 10^7^ CFU/mL, and the log mean was 1.05 log10. There was a decrease of 3.56X10^8^ CFU/mL between pre-asepsis and post-asepsis quantifications, and the logarithmic reduction was 6.46 ± 2.62 log10 (Fig. 1).

### Bacterial quantification in MRSA agar

Approximately 39% (18/46) of the samples corresponding to the pre-asepsis swabs showed bacterial growth in MRSA medium. Also, none of the swabs harvested at post-asepsis after the two protocols showed bacterial growth in this medium. Subsequently, isolates were identified, being observed that only one isolate was confirmed to be *S. aureus* and one isolate as *S. epidermidis*. The false positive cultures corresponded to other members of the genus *Staphylococcus* (*S. capitis*, *S. saprophyticus*, *S. xylosus* and *S. sciuri*) and of the Enterobacteriaceae family (*Enterobacter cloacae*, *Proteus* sp., *Klebsiella* sp., *Klebsiella pneumoniae* and *Serratia* sp.). *Corynebacterium pseudodiphteriticum*, *Cellulomonas* sp. and *Enterococcus* sp. were also found, and filamentous fungi were detected in one sample.

### Statistics

**Fig. 2:**
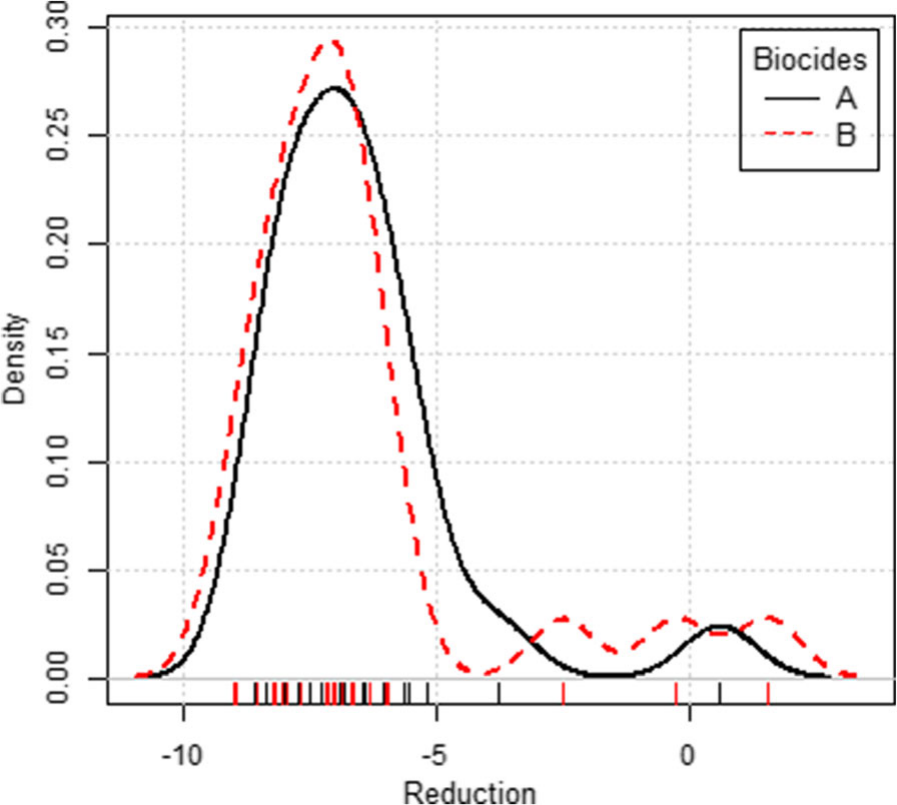
DensityPlot plot corresponding to the logarithmic reduction distribution for the two protocols. The distribution curve of the 23 log reductions for povidone-iodine (A) and chlorhexidine protocols (B)

Statistical analysis using ANOVA test with repeated measures showed that there was no significant difference between the logarithmic reduction of bacterial quantification relative to the pre- and post-asepsis in both groups (*p* value > 0.05) (Fig. 1), and the distribution curve of the log reductions was very similar for both agents (Fig. 2).

## Discussion

A SSI is defined as a post-surgery infection in the incisional superficial or deep tissue, including organs, which occurs in the first 30 days after the procedure [15]. According to WHO, SSI are directly related with extended hospitalization and higher healthcare costs, as well as with increased morbidity and mortality rates, in both human and veterinary medicines [16, 17]. These infections can be a result of surgical bacterial contamination, with the animals’ immune system and the bacteria virulence potential playing important roles in SSI development and severity [15]. In a surgical context, skin asepsis is one of the most important preventive measures of SSI, targeting the elimination or a major reduction of the skin transient microbiota, preventing its multiplication in the short term (i.e. hours) [6, 18].

In this study, the clear majority of the samples evaluated did not present positive bacterial growth, both for the swabs collected at post-asepsis either with povidone-iodine (74%) or with chlorhexidine (70%), evidencing that both protocols were generally efficient. It is known that the currently available antiseptic products cannot eliminate all the microorganisms present in the skin, and approximately 20% of the skin microbiota of small animals’ remains protected in deeper strata and follicles, regardless of the asepsis protocol adopted [6]. As such, Food and Drug Administration (FDA) defines a skin disinfectant as a fast-acting agent with a broad spectrum of action and with persistent antiseptic power, capable of significantly reduce the number of microorganisms on intact skin [16].

Only approximately 9% of the samples did not present a significant logarithmic reduction relative to the bacterial quantification before and after asepsis, which may indicate resistance to antiseptic agents. These bacteria were isolated and cryopreserved for future characterization. To preserve the role of antiseptic agents in the control of infections, it is essential to prevent the emergence of bacteria resistant to these compounds, as well as cross resistance, promoting the appropriate use of these antimicrobial compounds [19].

Most pre-asepsis swabs (approximately 61%, 28/46 animals) and all post-asepsis swabs did not show bacterial growth in MRSA medium. Surprisingly, from the ones which allowed bacteria isolation, none of the isolate was identified as *S. pseudintermedius*, the most prevalent agent isolated from surgical site infections in dogs, and thus none MRSP was detected. Only one *S. aureus* and one *S. epidermidis* were detected in MRSA medium (4.3%, 2/46 animals). Therefore, only 4.3% animals had methicillin-resistant species. Chromogenic agar media are selective for MRSA and are widely used, since they are easy to interpret, although they may present false-positive results. Different authors have concluded that most false positives in MRSA media are staphylococci [20–22], but other microorganisms can be found, such as *Proteus*, *Corynebacterium* and *Micrococcus* [22]. Filamentous filaments fungi can also be isolated in chromogenic medium ChromID MRSA [20]. In accordance, in our study other bacterial species resistant to methicillin have grown in MRSA medium (34.8%, 16/46 animals), mainly other staphylococci as well as Enterobacteriaceae. According to Michael et al., 2015 [21], the use of a selective chromogenic agar medium was shown to be non-specific for the presumptive diagnosis of the presence of MRSA in canine skin. As such, the combined use of two selective media for MRSA or the combined use of other methods for identifying these agents should be performed, aiming at increasing detection specificity [21]. In the present study, Chapman agar was not used because our aim was studying the effect of biocides in methicillin-resistant species from clinical samples, and not in staphylococci in general. The use of blood agar was not considered for the same reason, and the use of MacConkey agar was discarded as staphylococci do not grow in this medium. MRSA medium results were confirmed by PCR, by detection of the *mecA* gene in all staphylococci performed as previously described [23], confirming the absence of methicillin-resistant bacterial species.

Although the sample size in this study is not high (46 dogs), it should not be neglected. No statistically significant differences (*p* > 0.05) were observed in the logarithmic reduction of the bacterial quantification between samples collected at pre-asepsis and post-asepsis times in both protocols (Figs. 1 and 2), allowing to conclude that the efficacy of both antiseptic solutions tested were similar. These results are in line with the ones of Osuna et al.*,* 1990 [5], which compared three pre-surgical skin preparation protocols of dogs using povidone-iodine, 4% chlorhexidine gluconate with saline and 70% isopropyl alcohol rinse, and concluded that they were equally effective.

The combination of alcohol and chlorhexidine gluconate allows to combine the immediate antimicrobial effect of the alcohol to the effect of chlorhexidine, resulting in a superior antiseptic efficacy [24]. Likewise, the combination of isopropyl alcohol with an iodophor allows to obtain a product with immediate effectiveness, requiring a shorter application period when compared to the iodophors individually [25]. Although an alcoholic solution of povidone-iodine was not evaluated in this study, a previous work by Gibson et al., 1997 [4] had already concluded that a one-step iodophor skin preparation solution (0.7% available iodine in isopropyl alcohol - DuraPrep) was as effective in pre-surgical skin antisepsis as chlorhexidine gluconate solution followed by alcohol.

According to Cronquist et al., 2001 [26], the log means of bacteria on the skin of humans varies significantly between anatomical regions, being much lower than the one from the skin of canids. The differences in the bacterial load on the skin of dogs and humans may explain why in our study the mean log reduction in the skin bacterial load after application of either protocol is above that the one specified by the Tentative Final Monograph published by the FDA in 1994.

The higher mean of bacterial quantification in dogs’ skin appears to be a greater challenge when compared to humans, since dogs have thicker coating, low hygiene frequency and contact with a more contaminated environment. Therefore, it is understandable that the techniques that are effective in humans may be less efficient in animals [4]. In fact, our results differ from the ones observed in human medicine, where it was concluded that alcoholic solutions of chlorhexidine had superior efficacy then povidone-iodine [27, 28], in accordance to WHO and CDC guidelines for human medicine [1, 2]. The different efficiency between biocides in human and veterinary settings allows to hypothesize that there is probably a limiting bacterial load, from which the presence of alcohol in skin asepsis protocols will not improve its efficacy for application to dogs.

Regarding SSI development, all animals in our study were examined by the veterinary surgeon 24 h and 30 days after surgery. Despite being previously stated that there is a high risk for SSI development if the skin bacterial load at the surgery region is higher than 10^5^ CFU/mL [15], in our study none of the animals developed SSI, revealing the efficiency of the prophylactic therapeutic protocols performed.

## Conclusions

Studies on pre-surgical skin asepsis protocols with application in veterinary medicine are scarce, and the available protocols using povidone-iodine or chlorhexidine biocides are the two most frequently used in both human and veterinary surgery. According to our study, the use of 7.5% povidone-iodine or an alcoholic solution of 2% chlorhexidine appears to have similar efficacy in reducing the total load of skin bacteria, including methicillin-resistant bacterial species present on the skin and preventing surgical site infections in dogs undergoing surgery.

## Abbreviations

CDC: Center for Disease Control and Prevention
FDA: Food and Drug Administration
MRSA: Methicillin-resistant *S. aureus*
MRSP: Methicillin-resistant *S. pseudintermedius*
SSI: Surgical site infections
WHO: World Health Organization
